# Efficacy and Safety of Canakinumab Compared With Standard Therapy for the Treatment of Non‐Small Cell Lung Cancer (NSCLC): A Systematic Review

**DOI:** 10.1002/cnr2.70627

**Published:** 2026-07-22

**Authors:** Kamiar Izadpanah, Masoud Rezaei, Seyed Aria Nejadghaderi, Hamid Sharifi

**Affiliations:** ^1^ HIV/STI Surveillance Research Center, and WHO Collaborating Center for HIV Surveillance, Institute for Futures Studies in Health Kerman University of Medical Sciences Kerman Iran; ^2^ Research Center for Hydatid Disease in Iran Kerman University of Medical Sciences Kerman Iran; ^3^ Knowledge Hub for Migrant and Refugee Health, Institute for Futures Studies in Health Kerman University of Medical Sciences Kerman Iran

**Keywords:** canakinumab, carcinoma, Interleukin‐1, non‐small cell lung neoplasm, systematic review, treatment outcome

## Abstract

**Background:**

Lung cancer remains a leading cause of cancer‐related mortality worldwide, with few effective treatment options available for advanced stages. Canakinumab, an IL‐1β inhibitor, has been investigated as a potential therapeutic agent due to its role in modulating tumor‐promoting inflammation. However, its clinical efficacy and safety in NSCLC (non‐small cell lung cancer) continue to be uncertain.

**Aims:**

We aimed to evaluate the efficacy and safety of canakinumab compared with other standard therapies in NSCLC treatment.

**Methods and Results:**

A systematic review was conducted in accordance with PRISMA guidelines. Four electronic databases (PubMed, Scopus, Web of Science, and Embase) were searched for clinical trials assessing canakinumab for NSCLC. Eligible studies included phase I–III clinical trials. The Synthesis Without Meta‐analysis (SWiM) guidelines were followed due to substantial heterogeneity in studies. Outcomes evaluated included objective response rate (ORR), progression‐free survival (PFS), overall survival (OS), major pathologic response (MPR), and treatment‐related adverse events.

Five clinical trials (three phase III, one phase II, and one phase IB) met the inclusion criteria, with sample sizes ranging from 88 to 1382 patients. The included studies evaluated canakinumab in four distinct clinical settings: adjuvant therapy after complete resection (CANOPY‐A), first‐line treatment in combination with pembrolizumab plus chemotherapy (CANOPY‐1) or spartalizumab plus chemotherapy, second‐line treatment in combination with docetaxel (CANOPY‐2), and neoadjuvant therapy with or without pembrolizumab (CANOPY‐N). Compared to standard therapies, canakinumab did not significantly improve ORR, PFS, OS, or MPR across any clinical setting. Safety analysis showed comparable adverse event rates between intervention and control groups.

**Conclusion:**

Despite its promising mechanistic rationale, Canakinumab did not show significant clinical benefits in NSCLC. Although it is generally well tolerated, its role in NSCLC treatment remains uncertain. Future research should concentrate on identifying predictive biomarkers and optimizing combination strategies to enhance its therapeutic potential.

## 1 Introduction

Lung cancer remains the leading cause of cancer‐related mortality worldwide, accounting for an estimated 1.8 million deaths annually [1]. Histologically, non‐small cell lung cancer (NSCLC) constitutes approximately 85% of all lung cancer cases and is subclassified into adenocarcinoma, squamous cell carcinoma, and large cell carcinoma, each with distinct molecular profiles, clinical behavior, and therapeutic implications [2]. The past decade has seen transformative advances in the treatment landscape of advanced NSCLC, driven by immune checkpoint inhibitors targeting the PD‐1/PD‐L1 axis and molecularly targeted therapies against actionable driver alterations including EGFR mutations, ALK rearrangements, ROS1 fusions, BRAF V600E mutations, and KRAS G12C mutations [3, 4]. Despite these therapeutic innovations, five‐year survival rates for metastatic NSCLC remain below 10%, underscoring the persistent need for novel therapeutic strategies targeting alternative mechanisms of tumor progression and immune evasion [5].

Interleukin‐1β (IL‐1β), a pro‐inflammatory cytokine, has emerged as a factor in the pathogenesis and progression of lung cancer [6]. IL‐1β contributes to tumor growth and metastasis through multiple mechanisms, which makes it a potential therapeutic target. One of its primary roles is in promoting angiogenesis. IL‐1β induces the production of vascular endothelial growth factor (VEGF), a mediator of angiogenesis, thereby facilitating the growth and survival of cancer cells [7]. Additionally, IL‐1β enhances tumor invasiveness by stimulating the secretion of matrix metalloproteinases, enzymes that degrade the extracellular matrix and enable cancer cells to invade surrounding tissues [8]. Beyond its direct effects on tumor cells, IL‐1β also shapes the tumor microenvironment (TME) by recruiting immunosuppressive cells such as myeloid‐derived suppressor cells and tumor‐associated macrophages [9]. These cells suppress anti‐tumor immune responses and create an environment conducive to tumor progression [10].

IL‐1β activates the NF‐κB pathway, leading to the transcription of genes involved in cell survival, proliferation, and inflammation, further contributing to tumor growth [11]. It also plays a role in inducing epithelial‐to‐mesenchymal transition that changes the metastatic potential of cancer cells [12]. Recent studies have highlighted the interaction between IL‐1β and immune checkpoints, suggesting that IL‐1β may upregulate the expression of programmed death‐ligand 1 (PD‐L1) on tumor cells [13]. This upregulation contributes to immune evasion by inhibiting T‐cell activity, enabling tumors to escape immune surveillance.

Canakinumab, a human monoclonal antibody targeting IL‐1β, belongs to the class of biologic therapies designed to modulate inflammatory pathways. Unlike anakinra, a recombinant IL‐1 receptor antagonist that requires daily subcutaneous administration due to its short half‐life, canakinumab exhibits a longer half‐life, allowing dosing every 3 weeks, which enhances patient convenience and adherence [14, 15]. This distinction in pharmacokinetics is critical, as canakinumab's sustained inhibition of IL‐1β suppresses inflammatory signaling more than the transient blockade of anakinra. Furthermore, canakinumab's specificity for neutralizing IL‐1β, rather than broadly targeting IL‐1 receptors, may reduce off‐target effects and improve therapeutic precision in inflammatory conditions [16].

In the Canakinumab Anti‐Inflammatory Thrombosis Outcomes Study (CANTOS), a cardiovascular trial, exploratory analyses revealed a dose‐dependent reduction in lung cancer incidence and mortality among participants receiving canakinumab, suggesting a potential anticancer effect mediated through IL‐1β inhibition [17]. Despite growing interest in targeting IL‐1β in lung cancer treatment, the therapeutic potential of canakinumab remains uncertain. Several clinical trials have explored its efficacy, yet findings have been inconsistent, with no significant survival benefit observed in subsequent studies. The current study aims to systematically evaluate the efficacy and safety of targeting this pathway with canakinumab compared with other standard therapies to better define its potential in improving clinical outcomes in NSCLC treatment.

## 2 Methods

### 2.1 Search Strategy

A literature search was conducted across four electronic databases: PubMed, Scopus, Web of Science, and Embase. The search covered all records from inception to May 13, 2026. The search query was designed using combinations of the terms “Canakinumab” and related terms, as well as “Lung cancer” along with its related terms. The search strategy, including specific search terms and syntax used for each database, is detailed in Table S1. No language restrictions were imposed during the electronic searches; records in all languages were considered for screening, with translation performed as needed for potentially eligible non‐English studies. Furthermore, no automated study‐type or publication‐type filters (e.g., clinical trial limits, human filters, or age restrictions) were applied to the database searches, in order to preserve maximum sensitivity for all clinically relevant study designs. In addition to searching the databases, we screened the first 300 results from Google Scholar [18]. We also performed backward and forward citation searching on the included studies to identify additional relevant articles.

### 2.2 Study Selection

Two authors (K.I. and M.R.) independently screened the retrieved records' titles and abstracts to determine their inclusion eligibility. Any disagreements between the two reviewers regarding the inclusion or exclusion of studies were resolved through discussion and, if necessary, by consultation with a third author (S.A.N.). The inclusion criteria were as follows: original clinical trial studies that evaluated canakinumab as an intervention in patients with NSCLC, regardless of histological subtype. No language restrictions were applied. Studies published in any language were considered for inclusion. Studies were excluded if they involved other IL‐1 inhibitors, or were animal studies, conference abstracts, or review articles. After the initial screening, the full texts of the potentially eligible studies were assessed to confirm their suitability for inclusion in the review.

### 2.3 Data Extraction

Data extraction was conducted systematically by two independent authors. The following information was collected from each included study: first author, study title, year of publication, country, study design, phase of the trial, study population and demographics, smoking status, follow‐up duration, intervention characteristics, lung cancer subtype, primary disease stage, survival metrics (e.g., overall survival [OS], progression‐free survival [PFS]), and adverse events.

### 2.4 Quality Assessment

The quality of the included studies was assessed using appropriate tools based on their study design. For randomized controlled trials (RCTs), the Cochrane Risk of Bias (RoB) 2 tool was employed to evaluate the risk of bias across various domains [19]. For non‐randomized studies, the Risk of Bias In Non‐randomized Studies of Interventions (ROBINS‐I) Version 2 tool was used [20]. The RoB 2 tool assesses bias across five domains: randomization process, deviations from intended interventions, missing outcome data, measurement of outcomes, and selection of reported results. Studies were categorized as having low, some concerns, or high risk of bias. The ROBINS‐I Version 2 tool evaluates non‐randomized studies across seven domains, including confounding, participant selection, classification of interventions, deviations from intended interventions, missing data, outcome measurement, and reporting bias. Each study was classified as having a low, moderate, serious, or critical risk of bias.

### 2.5 Data Synthesis

Due to substantial clinical and methodological heterogeneity across the included trials, a meta‐analysis was not performed, as statistical aggregation was precluded by several major domains of variation. Clinically, the trials differed across: (i) disease settings and stages, which spanned adjuvant therapy for completely resected early‐stage disease (CANOPY‐A), neoadjuvant therapy for resectable early‐stage disease (CANOPY‐N), first‐line therapy for treatment‐naïve advanced/metastatic disease (CANOPY‐1 and Santoro et al.), and second‐line therapy for pretreated advanced/metastatic disease (CANOPY‐2); (ii) treatment regimens, which ranged from canakinumab monotherapy to complex combinations with docetaxel, pembrolizumab, spartalizumab, or platinum‐doublet chemotherapy; and (iii) control groups, which utilized placebo, active chemotherapy monotherapy, or immunotherapy alone. Methodologically, the trials exhibited significant differences in study design (ranging from a Phase Ib non‐randomized trial to Phase III double‐blind RCTs) and divergent, mathematically non‐combinable primary endpoints (including disease‐free survival [DFS], OS, PFS, and major pathologic response [MPR]). Statistical pooling of these highly diverse settings and clinical endpoints would yield mathematically forced and clinically misleading summary estimates. Therefore, a narrative synthesis was conducted to systematically summarize the findings. The results were organized based on clinical settings to report the efficacy and safety of canakinumab in NSCLC. We used the Synthesis Without Meta‐analysis (SWiM) guidelines [21].

## 3 Results

### 3.1 Study Selection

**FIGURE 1:**
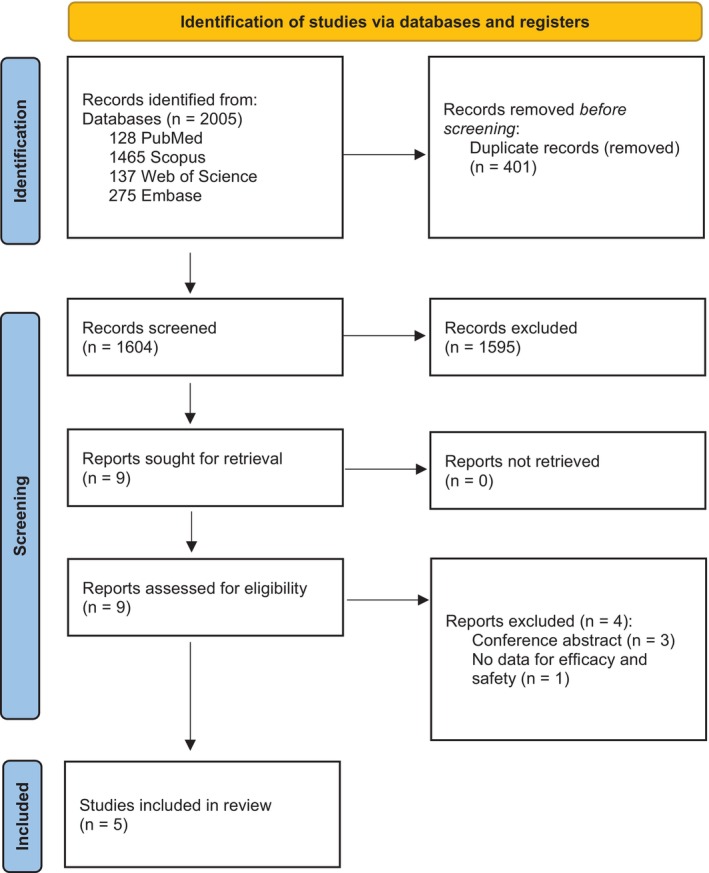
Study selection process.

A systematic literature search was conducted across four electronic databases, yielding 2005 records (128 from PubMed, 1465 from Scopus, 137 from Web of Science, and 275 from Embase). After removing 401 duplicate records, 1604 records remained for screening based on their titles and abstracts. Of these, 1595 records were excluded as they did not meet the inclusion criteria. The remaining nine full‐text articles were assessed for eligibility. Following a detailed evaluation, four articles were excluded, including three conference abstracts [22, 23, 24] and one for insufficient data for efficacy and safety assessments [25]. Moreover, no further studies were found through Google Scholar. Ultimately, five studies met the criteria and were included in this systematic review [14, 26, 27, 28, 29] (Figure 1).

### 3.2 Quality Assessment

**FIGURE 2:**
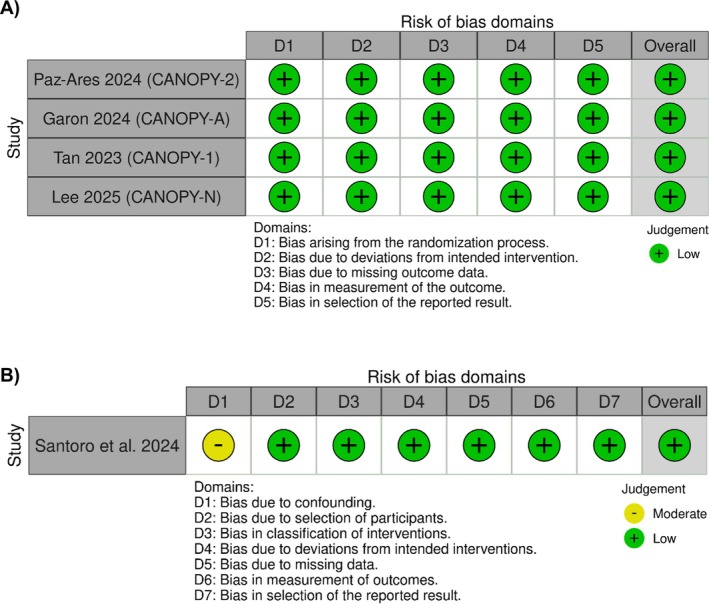
(A) Risk of bias assessment for randomized controlled trials using the Cochrane Risk of Bias version 2 tool. (B) Risk of bias assessment for the non‐randomized study using the ROBINS‐I tool.

For the four RCTs [14, 26, 27, 29], the RoB 2 tool was employed. All four RCTs demonstrated low risk of bias across all domains (Figure 2A). For the non‐randomized study [28], the ROBINS‐I Version 2 tool was used. There was a low risk of bias for all domains except for the domain 1, which is bias due to confounding, rated as moderate risk. Despite this single moderate risk rating, the overall quality assessment for Santoro et al. was determined to be low risk [28] (Figure 2B).

### 3.3 Study and Participants Characteristics

This systematic review included five multicenter studies: four RCTs [14, 26, 27, 29] and one open‐label clinical trial [28]. Three RCTs were phase III [14, 26, 27], one was phase II [29], and the open‐label study was phase IB [28]. The included studies were published in 2023–2025. The sample sizes varied considerably across studies, ranging from 88 to 1382 participants. The largest study was conducted by Garon et al. (*n* = 1382) [26], followed by Tan et al. (*n* = 643) [27], Paz‐Ares et al. (*n* = 237) [14], Santoro et al. (*n* = 111) [28], and Lee et al. (*n* = 88) [29]. All studies focused on patients with NSCLC but with different disease stages and prior treatment status. Paz‐Ares et al. included stage IIIB/IV patients post‐platinum‐based chemotherapy and immunotherapy [14]. Garon et al. enrolled resected stage II‐IIIA/IIIB patients post‐cisplatin therapy [26]. Tan et al. studied patients with advanced NSCLC and stable CNS metastases [27]. Santoro et al. recruited treatment‐naïve advanced NSCLC patients [28]. Lee et al. (the CANOPY‐N trial) enrolled patients with resectable stage IB–IIIA NSCLC in the neoadjuvant setting [29]. Treatment duration varied across studies, from 6 weeks (2 cycles) in Lee et al. [29], 14.8 weeks in Santoro et al. [28], 8.2 months in Tan et al. [27], and approximately 12 months in Garon et al. [26]. Follow‐up periods were reported in three studies [14, 27, 28], ranging from 33 weeks in Santoro et al. [28], 13.8 months in Paz‐Ares et al. [14] to 21.2 months in Tan et al. study [27], while Garon et al. did not report follow‐up duration [26], and Lee et al. focused on perioperative outcomes [29]. All studies excluded patients with EGFR/ALK alterations [14, 26, 27, 28, 29] (Table 1).

**TABLE 1:** Baseline characteristics of included studies evaluating Canakinumab in non‐small cell lung cancer treatment.

Study ID	Study design and blinding	Country	Phase	Sample size			Study population	Total number of males (%)	Age (mean [SD] or median [range])	Length of treatment	Follow‐up duration
				Total	Intervention	Control					
Paz‐Ares et al. [14]	RCT	Multicenter	III	237	120	117	Stage IIIB/IV NSCLC patients post‐PDC and PD‐1/PD‐L1 inhibitor therapy, with WHO performance status 0–1; EGFR/ALK alterations excluded.	82 (68%)	Intervention: 64 (58–70) Control: 63 (56–69)	Intervention: median 4.1 months (range, 1.4–6.3) Control: median 4.1 months (range, 1.5–6.9)	Median 13.8 months
Garon et al. [26]	RCT	Multicenter	III	1382	693	689	Resected stage II‐IIIA/IIIB NSCLC patients post‐cisplatin therapy; unresectable/metastatic cases excluded.	430 (62%)	63.0 (27–83)	Intervention: 11.7 [IQR: 6.2–12.4]; Control: 11.8 [IQR: 6.2–12.4] months	N/A
Tan et al. [27]	RCT	Multicenter	III	643	320	323	Advanced NSCLC, stable CNS metastases, and no prior immunotherapy or EGFR/ALK alterations.	227 (70.9%)	63 (57–69)	Intervention: 8.2 [IQR: 0.0–25]; Control: 7.7 [IQR: 0.0–25] months	21.2 (18.8–25.5) months
Santoro et al. [28]^a^	Open‐label clinical trial	Multicenter	IB	111	7	38	Treatment‐naïve adults with advanced NSCLC (ECOG PS 0–1, measurable tumors) lacking EGFR, ALK, or ROS1 alterations, excluding those with interstitial lung disease, leptomeningeal metastases, recent radiotherapy, or prior immunotherapy.	6 (85.7%)	63.0 (58.0–69.0)	14.8 weeks	Intervention: 33.0 (IQR: 24.0–53.9) Control: 44.4 (IQR: 18.0–86.9) weeks
Lee et al. [29]	RCT, open‐label	Multicenter	II	88	35 (CAN) 35 (CAN + PEM)	18 (PEM)	Resectable stage IB–IIIA NSCLC (neoadjuvant).	52 (59%)	67.0 (61.0–71.5)	6 weeks (2 cycles)	NA

Abbreviations: ALK: anaplastic lymphoma kinase; CAN: Canakinumab; ECOG: Eastern Cooperative Oncology Group; EGFR: epidermal growth factor receptor; IQR: interquartile range; N/A: not available; NSCLC: non‐small cell lung cancer; PD‐1: programmed death‐1; PDC: platinum‐based doublet chemotherapy; PD‐L1: programmed death‐ligand 1; PEM: pembrolizumab; ROS‐1: ROS proto‐oncogene 1.

^a^ The Santoro et al. study was a phase Ib open‐label trial with multiple treatment groups (A–E). For this systematic review, the intervention group (*n* = 7) comprised patients receiving spartalizumab + pemetrexed + cisplatin + canakinumab (Group E), and the control group (*n* = 38) comprised patients receiving spartalizumab + pemetrexed + cisplatin without canakinumab (Group B). The remaining 66 patients in other groups were excluded from comparative analysis due to differences in chemotherapy backbone precluding direct comparison.

### 3.4 Efficacy Outcomes

First‐line advanced/metastatic setting (CANOPY‐1 and Santoro et al.): The CANOPY‐1 trial [27], the largest study included (*n* = 643), evaluated canakinumab versus placebo in combination with first‐line pembrolizumab plus platinum‐based chemotherapy in patients with advanced NSCLC. ORR was nearly identical between groups: 45.6% (95% CI: 40.1–51.3) in the canakinumab arm versus 45.5% (95% CI: 40.0–51.1) in the placebo arm. Median PFS was 6.8 months in both groups, with a non‐significant HR of 0.85 (95% CI: 0.67–1.09). Median OS was 20.8 months (95% CI: 16.3–NE) versus 20.2 months (95% CI: 16.2–22.4), HR 0.87 (95% CI: 0.70–1.10). The disease control rate was 86.9% versus 84.8%, and median DoR was 14.3 versus 13.6 months [27].

The Santoro et al. phase Ib open‐label trial compared spartalizumab plus pemetrexed/cisplatin with or without canakinumab in treatment‐naive advanced NSCLC. ORR was 57.1% (95% CI: 18.4–90.1) in the canakinumab group versus 55.3% (95% CI: 38.3–71.4) in the control group. Median PFS was 7.5 months (95% CI: 4.1–12.4) versus 10.4 months (95% CI: 5.4–26.4) favoring the control group. Median OS was 21.0 months (95% CI: 4.8–NE) versus 29.7 months (95% CI: 17.8–39.9), also favoring the control group. Disease control rate was 100% versus 81.6%, and median DoR was 7.1 months versus 30.1 months. Neither study demonstrated a statistically significant benefit from the addition of canakinumab [28].

The CANOPY‐2 trial (*n* = 237) evaluated canakinumab plus docetaxel versus placebo plus docetaxel in patients with stage IIIB/IV NSCLC who had progressed after prior platinum‐based chemotherapy and immunotherapy. ORR was 15% (95% CI: 9.1–22.7) in the canakinumab group versus 14% (95% CI: 8.0–21.3) in the placebo group. Median PFS was 4.17 months (95% CI: 2.96–5.42) versus 4.21 months (95% CI: 3.06–5.13). Median OS was 10.55 months (95% CI: 8.15–12.39) versus 11.3 months (95% CI: 8.54–13.80), HR 1.06 (95% CI: 0.76–1.48; one‐sided *p* = 0.633). Disease control rate was 66% versus 62%, and median DoR was 4.1 months (95% CI: 2.9–11.2) versus 5.4 months (95% CI: 4.2–NE). The study did not meet its primary endpoint of OS [14].

The CANOPY‐A trial (*n* = 1382) evaluated canakinumab versus placebo as adjuvant therapy in patients with completely resected stage II‐IIIA/IIIB NSCLC. The primary endpoint was DFS. Median DFS was 35.0 months in the canakinumab group versus 29.7 months in the placebo group (HR: 0.94; 95% CI: 0.78–1.14), a non‐significant improvement. ORR was not reported due to the adjuvant setting. No survival benefit was observed with canakinumab [26].

The CANOPY‐N trial (*n* = 88) evaluated canakinumab alone, canakinumab plus pembrolizumab, or pembrolizumab alone as neoadjuvant therapy in patients with resectable stage IB–IIIA NSCLC. The primary endpoint was MPR. MPR was achieved by 2.9% (95% CI: 0.07–14.92) of patients in the canakinumab monotherapy arm, 17.1% (95% CI: 6.56–33.65) in the canakinumab plus pembrolizumab arm, and 16.7% (95% CI: 3.58–41.42) in the pembrolizumab alone arm, indicating no improvement with canakinumab‐containing regimens. Per RECIST v1.1, no patient in the canakinumab arm achieved an objective response, compared to 8.6% in the combination arm and 11.1% in the pembrolizumab arm. Notably, 95.5% of patients successfully underwent surgery, with 85.7% achieving resection within the prespecified window, demonstrating that canakinumab did not adversely affect surgical outcomes [29] (Table 2).

**TABLE 2:** Efficacy outcomes of Canakinumab in non‐small cell lung cancer treatment across included studies.

Study ID	Objective response rate	Disease control rate	Progression‐free survival	Overall survival	Duration of response	Disease free survival
Paz‐Ares et al. [14]	Intervention: 18 (15% [9.1–22.7]) Control: 16 (14% [8.0–21.3])	Intervention: 79 (66% [56.6–74.2]) Control: 72 (62% [52.1–70.4])	Intervention: 4.17 months (2.96–5.42) Control: 4.21 months (3.06–5.13)	Intervention: 10.55 months (8.15–12.39) Control: 11.3 months (8.54–13.80)	Intervention: 4.1 (2.9–11.2) Control: 5.4 (4.2 to NE)	N/A
Garon et al. [26]	N/A	N/A	N/A	Intervention: OS HR, 0.72; 95% CI, 0.48 to 1.08 Control: N/A	N/A	Intervention: Median: 35.0 months HR, 0.94; 95% CI, 0.78 to 1.14 Control: Median: 29.7 months HR, 0.94; 95% CI, 0.78 to 1.14
Tan et al. [27]	Intervention: 45.6% (40.1 to 51.3) Control: 45.5% (40.0 to 51.1)	Intervention: 86.9% (82.7–90.4) Control: 84.8% (80.4–88.6)	Intervention: 6.8 months (5.6 to 7.8); HR, 0.85; 95% CI, 0.67 to 1.09 Control: 6.8 months (5.5 to 6.9)	Intervention: 20.8 months (16.3‐NE); HR, 0.87; 95% CI, 0.70 to 1.10 Control: 20.2 months (16.2–22.4)	Intervention: 14.3 (10.40 to NE) months Control: 13.6 (10.30 to NE) months	N/A
Santoro et al. [28]	Intervention: 4 (57.1%; 18.4–90.1) Control: 21 (55.3%; 38.3–71.4)	Intervention: 7 (100%; 59.0–100) Control: 31 (81.6%; 65.7–92.3)	Intervention: 7.5 (4.1–12.4) months Control: 10.4 (5.4–26.4)	Intervention: 21 (4.8‐NE) months Control: 29.7 (17.8–39.9) months	Intervention: 7.1 (1.4‐NE) months Control: 30.1 (9.0‐NE) months	N/A
Lee et al. [29]	CAN (0%)|CAN + PEM (8.6%)|PEM (11.1%)^a^	N/A	N/A	N/A	N/A	N/A

*Note:* Disease control is defined as objective response + stable disease.

Abbreviations: CAN: Canakinumab; CI: confidence interval; HR: hazard ratio; N/A: Not available; NE: not estimated; PEM: Pembrolizumab.

^a^ Complete or partial response per RECIST v1.1 criteria.

### 3.5 Safety

Across the five studies reviewed, the overall incidence of treatment‐related adverse events was high and comparable between the canakinumab (intervention) and control groups. In the CANOPY‐N trial [29], any‐grade AEs occurred in 88.6% (canakinumab), 91.4% (combination), and 83.3% (pembrolizumab). Grade 3/4 AEs occurred in 28.6%, 25.7%, and 16.7%, respectively. Grade 5 AEs occurred in 8.6% (canakinumab), 2.9% (combination), and 5.6% (pembrolizumab); all six on‐treatment deaths occurred postoperatively and none were treatment‐related. No treatment‐related grade 5 AEs were reported in any arm. Paz‐Ares et al. reported 88% in the intervention arm versus 85% in controls [14] for any‐grade adverse events. Tan et al. observed 95.0% versus 93.8% [27], Garon et al. recorded 38.9% versus 31.5% [26], and Santoro et al. noted 100% incidence in both groups [28].

For grade 3/4 adverse events, a trend for higher incidence in the intervention group was seen in Paz‐Ares et al. (51% vs. 42%) [14], Tan et al. (57.2% vs. 51.9%) [27], and Garon et al. (6.4% vs. 2.9%) [26]. Santoro et al. demonstrated a lower rate in the intervention group compared to controls (42.9% vs. 60.5%) [28]. For grade 5 adverse events, the canakinumab group had higher rates in Paz‐Ares et al. (3% vs. 1%) and Santoro et al. (14.3% vs. 7.9%) [14, 28]. In contrast, Garon et al. reported no events in the intervention group compared to 0.3% in controls [26], and Tan et al. showed a lower rate with canakinumab (3.8% vs. 5%) [27]. Specific grade 1 and 2 adverse events were not reported in any studies (Table 3).

**TABLE 3:** Incidence of treatment‐related adverse events by severity grade in studies evaluating Canakinumab for non‐small cell lung cancer treatment.

Study ID	Any grade		Grade 3/4		Grade 5	
	Intervention	Control	Intervention	Control	Intervention	Control
Paz‐Ares et al. [14]	105 (88%)	97 (85%)	61 (51%)	48 (42%)	3 (3%)	1 (1%)
Garon et al. [26]	269 (38.9%)	217 (31.5%)	44 (6.4%)	20 (2.9%)	0 (0%)	2 (0.3%)
Tan et al. [27]	304 (95.0%)	302 (93.8%)	183 (57.2%)	167 (51.9%)	12 (3.8%)	16 (5%)
Santoro et al. [28]	7 (100%)	38 (100%)	3 (42.9%)	23 (60.5%)	1 (14.3%)	3 (7.9%)
Lee et al. [29]	CAN: 88.6%|CAN + PEM: 91.4%	PEM: 83.3%	CAN: 28.6%|CAN + PEM: 25.7%	PEM: 16.7%	CAN: 8.6%|CAN + PEM: 2.9%	PEM: 5.6%

Infection‐related adverse events were evaluated across all five studies, with notable differences observed by clinical setting and patient population. In CANOPY‐2 (second‐line advanced setting), infections of any grade were numerically higher in the canakinumab arm (49% vs. 41%), with grade 5 infections occurring in 7% versus 2% of patients; the most commonly reported infections were pneumonia, respiratory tract infection, and urinary tract infection. Treatment‐related deaths in the canakinumab arm included sepsis, pulmonary sepsis, and septic shock [14]. In CANOPY‐1 (first‐line advanced setting), infection rates were comparable between arms (any grade: comparable; grade 3/4: 15.0% vs. 12.4%; grade 5: 5.6% vs. 5.3%), and neutropenia grade 3/4 was higher in the canakinumab arm (24.4% vs. 16.8%) [27]. In CANOPY‐A (adjuvant setting), the incidence of infections was comparable between groups, with grade 3/4 infections in 3.3% versus 2.8% and on‐treatment deaths due to infections in < 1% in both arms [26]. In CANOPY‐N (neoadjuvant setting), infections of any grade occurred in 31.4% (canakinumab), 14.3% (canakinumab + pembrolizumab), and 38.9% (pembrolizumab alone); grade 3/4 infections were reported in 8.6%, 5.7%, and 5.6%, respectively; one grade 5 infection (pneumonia) occurred in the canakinumab monotherapy arm, and one death due to COVID‐19 infection occurred in the pembrolizumab arm. Neutropenia was reported in 20% of patients in the canakinumab arm (grade 3/4: 2.9%) [29]. In the Santoro et al. study, infections were the most frequent adverse event of special interest in the canakinumab‐containing group (Group E), occurring in 5 of 7 patients (71.4%); one death due to pneumonia was reported [28].

Collectively, these data suggest that the risk of infection with canakinumab may be influenced by the clinical context: higher in heavily pretreated populations (CANOPY‐2) and lower in treatment‐naive or early‐stage settings (CANOPY‐A, CANOPY‐1). Neutropenia rates were also higher in the canakinumab arms across studies where chemotherapy was administered concomitantly (Table 3).

## 4 Discussion

The findings of this systematic review highlighted the limited efficacy and notable safety profile of canakinumab in the treatment of NSCLC. Across the five included studies, canakinumab did not significantly improve key efficacy outcomes such as ORR, PFS, and OS compared to control groups. For instance, ORR was nearly identical between intervention and control arms in the most prominent trial by Tan et al. (45.6% vs. 45.5%) [27]. In comparison, Santoro et al. reported slightly higher ORR in the intervention group (57.1% vs. 55.3%) [28]. Similarly, PFS and OS outcomes showed no statistically significant differences, with some studies even suggesting a trend toward better outcomes in the control groups, as seen in Santoro et al., where OS was longer in the control arm (29.7 vs. 21 months) [28]. These results suggest that canakinumab may not provide a meaningful clinical benefit in terms of efficacy for NSCLC patients. However, the safety profile of canakinumab was generally favorable, with adverse events being comparable between intervention and control groups across all studies. While the incidence of any‐grade adverse events was high in both groups, grade 3/4 adverse event rates were slightly higher in the canakinumab arms in most studies, except for Santoro et al., which reported a lower rate in the intervention group. Grade 5 events were rare but slightly more frequent in the canakinumab groups in some studies, such as Paz‐Ares et al. (3% vs. 1%) [14]. Overall, the safety data suggest that canakinumab is well‐tolerated, but its limited efficacy raises questions about its role in NSCLC treatment.

The discrepancy between the promising signal from CANTOS and the uniformly negative results in oncology trials warrants careful consideration [25]. Several factors may explain this paradox. First, the CANTOS trial evaluated canakinumab in a secondary cardiovascular prevention population without established malignancy, where IL‐1β inhibition likely acted on early, inflammation‐driven carcinogenic processes—tumor initiation and promotion [30]. In contrast, the CANOPY program enrolled patients with established NSCLC, where multiple redundant oncogenic pathways are concurrently activated and may sustain tumor progression despite IL‐1β neutralization. Second, preclinical evidence suggests that IL‐1β plays a more prominent role in early tumorigenesis—angiogenesis, immune evasion, and epithelial‐to‐mesenchymal transition—than in established, late‐stage disease. By the time NSCLC is clinically detectable, the tumor may have evolved beyond IL‐1β dependence through activation of parallel signaling cascades (EGFR, KRAS, PI3K/AKT, MEK/ERK) that can bypass single‐cytokine blockade. Third, the tumor microenvironment in advanced NSCLC employs multiple overlapping immunosuppressive mechanisms—including regulatory T cells, M2‐polarized macrophages, and myeloid‐derived suppressor cells—that may not be adequately reversed by attenuating IL‐1β alone. These considerations highlight the fundamental distinction between intercepting tumor initiation and treating established malignancy, and suggest that successful targeting of the IL‐1β pathway may require intervention at earlier disease stages or in biologically selected patient populations [31].

The molecular pathway of IL‐1β in NSCLC treatment has garnered significant attention due to its role in tumor‐promoting inflammation and its impact on the TME [32]. IL‐1β, a pro‐inflammatory cytokine, is implicated in cancer progression by promoting immune suppression and facilitating the infiltration of immunosuppressive cells such as myeloid‐derived suppressor cells and tumor‐associated macrophages into the TME [33]. Preclinical studies have demonstrated that IL‐1β blockade using inhibitors like canakinumab and gevokizumab can remodel the TME, reducing tumor growth and enhancing antitumor immunity. For instance, in humanized mouse models of NSCLC, combining canakinumab with PD‐1 inhibitors significantly inhibited tumor growth and increased CD8+ T cell infiltration, suggesting a shift toward a less immunosuppressive TME [34, 35]. These findings are supported by clinical evidence from the CANTOS trial, where canakinumab reduced NSCLC incidence and mortality in atherosclerosis patients, highlighting the therapeutic potential of IL‐1β inhibition in NSCLC [25]. Targeting the IL‐1β pathway is promising for cancer immunotherapy, especially when combined with other immunomodulatory agents, to boost antitumor immune responses and enhance clinical outcomes.

The CANOPY‐1 trial, which evaluated canakinumab combined with pembrolizumab and platinum‐based chemotherapy as a first‐line treatment for advanced NSCLC, did not significantly improve PFS or OS compared to placebo [27]. While the safety profile was consistent, limitations included a short follow‐up period and no unexpected safety signals. Similarly, the CANOPY‐2 trial, investigating canakinumab with docetaxel for pretreated NSCLC patients, also failed to show a survival benefit, likely due to the heavily pretreated patient population and heterogeneity [14]. Both studies highlighted the importance of patient stratification and biomarker identification, although neither showed clear clinical efficacy for canakinumab in these settings. On the other hand, pembrolizumab remains a cornerstone of treatment, with checkpoint inhibitors generally showing survival benefits [36, 37, 38]. Nevertheless, integrating canakinumab into such regimens requires further research to optimize combination strategies and identify subgroups likely to benefit. Overall, the potential role of canakinumab in NSCLC treatment remains inconclusive but deserves further exploration.

Canakinumab as neoadjuvant therapy, alone or in combination with pembrolizumab, has been explored in the CANOPY‐N trial, aiming to improve outcomes for patients with resectable NSCLC [29].

Neoadjuvant therapies are crucial for increasing the chances of surgical success and reducing recurrence, especially in early‐stage NSCLC (stage IB‐IIIA) [39]. The lack of improvement in MPR rates with canakinumab, whether alone or in combination, adds to the growing body of evidence that IL‐1β inhibition does not provide meaningful clinical benefit across various NSCLC settings, including the neoadjuvant context.

The consistent failure of IL‐1β inhibition across unselected NSCLC populations underscores the critical need for predictive biomarkers to guide patient selection in future trials. While exploratory analyses from the CANOPY program identified baseline CRP levels and circulating tumor DNA detection as strong prognostic factors, neither biomarker demonstrated a differential predictive effect with respect to canakinumab treatment. CRP, as a downstream effector of IL‐1β signaling, remains a logical candidate for patient stratification, yet its utility as a predictive rather than prognostic biomarker requires prospective validation. Additional candidate approaches include: (i) tumor‐based immune microenvironment profiling to identify IL‐1β‐driven inflammatory signatures; (ii) assessment of NLRP3 inflammasome activation status; (iii) characterization of myeloid cell polarization states in the tumor microenvironment; and (iv) serial monitoring of inflammatory markers during treatment to identify early pharmacodynamic responses. The integration of these biomarker strategies into adaptive trial designs with pre‐specified interim analyses and go/no‐go decision criteria would enable more efficient evaluation of canakinumab in biologically defined subgroups.

### 4.1 Limitations

This study has several limitations. First, the systematic review included only five studies, which restricts our ability to replicate and confirm these clinical findings across independent, parallel cohorts within the same setting. However, the robustness of our overall conclusions is bolstered by a large cumulative sample size (*N* = 2461 patients) and the presence of three rigorously conducted, low risk‐of‐bias Phase III multicenter RCTs, which provide high statistical power and strong internal validity for their specific clinical settings. Second, the small sample size of some studies, such as Santoro et al. (*n* = 111) [28], further restricts the robustness of the findings in early‐phase evaluations. Third, the substantial clinical and methodological heterogeneity across the included trials (varying disease stages, treatment backbones, and control arms) precludes generalization regarding canakinumab's therapeutic efficacy across NSCLC as a whole. The generalizability (applicability to clinical practice) of our findings must therefore be used and restricted to the specific scenarios evaluated (e.g., adjuvant canakinumab monotherapy post‐cisplatin, or first‐line combinations with pembrolizumab and chemotherapy). Consequently, our findings may not generalize to other unstudied therapeutic combinations, different chemotherapy backbones, or biologically selected patient subpopulations. Fourth, excluding patients with EGFR/ALK alterations in all studies limits the applicability of the findings to this subgroup of NSCLC patients, who represent a significant proportion of the population. Fifth, our search strategy lacked database‐specific controlled vocabulary (e.g., MeSH) and did not explicitly include certain disease‐specific acronyms like “NSCLC” in the primary search string. Although the highly specific nature of the drug term “canakinumab” limits the risk of missed trials, this remains a methodological limitation. Sixth, because a quantitative meta‐analysis was not performed, we did not construct a traditional quantitative GRADE assessment. Seventh, we did not systematically search clinical trial registries (e.g., ClinicalTrials.gov, WHO ICTRP) for unpublished, ongoing, or negative‐result trials. Although our primary database searches, supplemented by Google Scholar and citation tracking successfully identified all known published trials, the absence of a formal registry search introduces a potential risk of publication bias. We cannot entirely exclude the possibility of unreported neutral or negative findings that might influence the overall evidence base. Future updates of this review should incorporate mandatory registry searches to provide a more comprehensive and bias‐minimized assessment. Eighth, this systematic review was not prospectively registered in PROSPERO or any other publicly available review registry. Although we adhered strictly to PRISMA 2020 reporting guidelines and employed dual‐independent reviewer processes to ensure methodological rigor, the absence of prospective registration may reduce transparency and increase the risk of unreported post hoc methodological changes. We acknowledge this as a limitation and recommend that future updates of this evidence synthesis be prospectively registered to enhance credibility and reproducibility. These limitations underscore the need for further research with larger, more homogeneous populations to better understand the role of canakinumab in NSCLC treatment.

## 5 Conclusion

Current evidence does not support routine use of canakinumab in NSCLC. Despite its promising molecular mechanism in targeting the IL‐1β pathway and preclinical evidence supporting its role in modulating the tumor microenvironment, five clinical trials across adjuvant, first‐line, second‐line, and neoadjuvant settings have consistently shown no significant improvement in ORR, PFS, or OS with canakinumab‐containing regimens compared to standard therapy. The safety profile of canakinumab is generally acceptable, with adverse event rates comparable to control groups; however, the risk of infection, particularly in heavily pretreated populations, warrants consideration. Future investigation, if pursued, should focus on biologically selected populations identified through prospective biomarker validation of inflammatory and molecular profiles rather than unselected broad clinical settings. Such trials should employ adaptive designs with built‐in biomarker validation, interim futility analyses, and pre‐specified go/no‐go criteria. Until such evidence emerges from well‐designed clinical trials, canakinumab has no established role in the routine management of NSCLC.

## Author Contributions

**Seyed Aria Nejadghaderi:** writing – review and editing, writing – original draft, conceptualization, methodology, visualization, resources, supervision, project administration, validation. **Hamid Sharifi:** writing – original draft, writing – review and editing, conceptualization, project administration, supervision, resources, methodology, formal analysis, investigation. **Masoud Rezaei:** writing – original draft, writing – review and editing, methodology, software, data curation, validation, investigation. **Kamiar Izadpanah:** conceptualization, writing – original draft, writing – review and editing, methodology, software, data curation, resources, formal analysis, validation, investigation.

## Funding

The authors have nothing to report.

## Disclosure

The authors used the AI tool ChatGPT to assist with language refinement. All authors read and confirmed the content of the manuscript.

## Ethics Statement

The authors have nothing to report.

## Consent

The authors have nothing to report.

## Conflicts of Interest

The authors declare no conflicts of interest.

## Supporting information


**Table S1:** Search strategies for PubMed, Scopus, Web of Science, Embase, and Google Scholar.

## Data Availability

The data that support the findings of this study are available in the Supporting Information of this article.
