# Supplementary material for: Efficacy and Safety of Canakinumab Compared With Standard Therapy for the Treatment of Non‐Small Cell Lung Cancer (NSCLC): A Systematic Review
Source: Cancer Rep (Hoboken). 2026 Jul 22;9(7):e70627. doi: 10.1002/cnr2.70627 (PMC13391215; doi:10.1002/cnr2.70627)
Supplement: Supplementary file 1 — Table S1: Search strategies for PubMed, Scopus, Web of Science, Embase, and Google Scholar. [file CNR2-9-e70627-s001.docx]

**Table S1.** Search strategies for PubMed, Scopus, Web of Science, Embase, and Google Scholar.

| **Database**  **(search date)** | **Step** | **Search strategy** | **Number of results** |
| --- | --- | --- | --- |
| PubMed  (5.13.2026) | #1 | ("Canakinumab"[All Fields] OR "Ilaris"[All Fields] OR "ACZ-885"[All Fields] OR "ACZ885"[All Fields] OR "immunoglobulin G1"[All Fields] OR "anti-(human interleukin 1beta) (human clone ACZ885 heavy chain V region)"[All Fields]) | 3,038 |
|  | #2 | (("Lung*"[All Fields] OR "Pulmonary"[All Fields]) AND ( "Neoplas*"[All Fields] OR "Cancer*"[All Fields] OR "Tumor*"[All Fields] OR "Tumour*"[All Fields] OR "Malignan*"[All Fields] OR "Carcinoma*"[All Fields] OR "Carcinoid*"[All Fields] OR "Adenocarcinoma*"[All Fields] OR "Lymphoma*"[All Fields] OR "Sarcoma*"[All Fields] OR "blastoma*"[All Fields] OR "Fibrosarcoma*"[All Fields] OR "Leiomyosarcoma*"[All Fields])) | 616,034 |
|  | #3 | #1 AND #2 | 128 |
| Scopus  (5.13.2026) | #1 | TITLE-ABS-KEY("Canakinumab" OR "Ilaris" OR "ACZ-885" OR "ACZ885" OR "immunoglobulin G1" OR "anti-(human interleukin 1beta) (human clone ACZ885 heavy chain V region)") | 29,298 |
|  | #2 | TITLE-ABS-KEY(("Lung*" OR "Pulmonary") AND ("Neoplas*" OR "Cancer*" OR "Tumor*" OR "Tumour*" OR "Malignan*" OR "Carcinoma*" OR "Carcinoid*" OR "Adenocarcinoma*" OR "Lymphoma*" OR "Sarcoma*" OR "blastoma*" OR "Fibrosarcoma*" OR "Leiomyosarcoma*")) | 885,320 |
|  | #3 | #1 AND #2 | 1,465 |
| Web of Science  (5.13.2026) | #1 | TS=("Canakinumab" OR "Ilaris" OR "ACZ-885" OR "ACZ885" OR "immunoglobulin G1" OR "anti-(human interleukin 1beta) (human clone ACZ885 heavy chain V region)") | 3,476 |
|  | #2 | TS=(("Lung*" OR "Pulmonary") AND ("Neoplas*" OR "Cancer*" OR "Tumor*" OR "Tumour*" OR "Malignan*" OR "Carcinoma*" OR "Carcinoid*" OR "Adenocarcinoma*" OR "Lymphoma*" OR "Sarcoma*" OR "blastoma*" OR "Fibrosarcoma*" OR "Leiomyosarcoma*")) | 649,363 |
|  | #3 | #1 AND #2 | 137 |
| Embase  (5.13.2026) | #1 | 'lung cancer'/exp OR 'ca lung' OR 'schneeberg disease' OR 'schneeberg lung disease' OR 'broncho-pulmonary cancer' OR 'bronchopulmonary cancer' OR 'cancer of the lung' OR 'cancer, lung' OR 'carcinogenesis of the lung' OR 'lung cancer' OR 'lung malignancies' OR 'lung malignancy' OR 'malignancies of the lung' OR 'malignancy of the lung' OR 'malignant lung neoplasm' OR 'malignant lung tumor' OR 'malignant neoplasm of the lung' OR 'malignant tumor of the lung' OR 'pulmonary cancer' OR 'pulmonary malignancies' OR 'pulmonary malignancy' | 669,981 |
|  | #2 | 'canakinumab'/exp OR 'acz 885' OR 'acz885' OR 'canakinumab' OR 'cmab 816' OR 'cmab816' OR 'ilaris' | 6,524 |
|  | #3 | #1 AND #2 | 275 |
| Google Scholar (1.24.2025) | #1 | "lung cancer" AND ("canakinumab" OR "acz 885") | About 3,610 |
